# T1 bladder carcinoma with variant histology: pathological features and clinical significance

**DOI:** 10.1007/s00428-021-03264-6

**Published:** 2022-02-04

**Authors:** Antonio Lopez-Beltran, Ana Blanca, Alessia Cimadamore, Rodolfo Montironi, Rafael J. Luque, Metka Volavšek, Liang Cheng

**Affiliations:** 1 Department of Morphological Sciences, University of Cordoba Medical School, Cordoba, Spain; 2 Maimonides Biomedical Research Institute of Cordoba, E-14004 Cordoba, Spain; 3 Institute of Pathological Anatomy and Histopathology, School of Medicine, Polytechnic University of the Marche Region, United Hospitals, Ancona, Italy; 4 UGC Anatomía Patológica, Hospital Universitario de Jaén, Jaén, Spain; 5 Institute of Pathology, Faculty of Medicine, University of Ljubljana, Ljubljana, Slovenia; 6 Departments of Pathology and Laboratory Medicine and Urology, School of Medicine, Indiana University, Indianapolis, IN USA

**Keywords:** Bladder, T1 urothelial carcinoma, Variant histology, Biomarker, Staging

## Abstract

The aim of the study was to stratify high-grade T1 (HGT1) bladder urothelial carcinoma into risk categories based on the presence of variant histology when compared to conventional urothelial carcinoma. The clinicopathological features of 104 HGT1 cases of urothelial carcinoma of the bladder with variant histology present in 34 (37%) were assessed. The endpoint of the study was disease-free survival and cancer-specific survival. Overall, variant histology was identified as a significant predictor of disease-free survival (*P* = 0.035). The presence of any specific variant histology (squamous, glandular, micropapillary, nested, microcystic, inverted growth, villous-like, basaloid, and lymphoepithelioma-like) was identified as a significant predictor of disease-free survival (*P* = 0.008) and cancer-specific survival (*P* = 0.0001) in HGT1 bladder cancer. Therefore, our results support including micropapillary HGT1 urothelial carcinoma within the aggressive high-risk category, as suggested by some recent clinical guidelines, but also favor nested, glandular, and basaloid to be placed in the high-risk category due to their potential of aggressive, life-threatening behavior and their limited response to bacillus Calmette-Guerin therapy. Conversely, the low-risk category would include urothelial carcinomas with squamous, inverted growth, or microcystic morphology, all with limited life-threatening potential and good response to current therapy. A very low-risk category would finally include patients whose tumors present villous-like or lymphoepithelioma-like morphology. In conclusion, our findings support the value of reporting the variant histology as a feature of variable aggressiveness in HGT1 urothelial carcinoma of the bladder.

## Introduction

Bladder carcinoma with variant histology represents about 20% of bladder urothelial carcinomas after TURBT (transurethral resection of bladder tumor) or cystectomy [1]. Most studies support variant histology as an aggressive feature with prognostic and therapeutic implications in advanced pT2-4 disease [2, 3].

On the other hand, after standardized BCG (bacillus Calmette-Guerin) therapy, up to 75% of high-grade T1 (HGT1) disease patients will experience tumor recurrence within 2 years; up to 25% of them with a high risk of progression to muscle-invasive disease [4, 5]. Hence, earlier identification of those tumors at risk of aggressive, life-threatening behavior would impact our practice. To accomplish this goal, it has been recommended that risk-associated features, such as tumor size, growth pattern and multifocality, extent/depth of invasion, concomitant urothelial carcinoma in situ, lymphovascular invasion, and variant histology, be reported [6]. The role of variant histology to stratify patients at risk in HGT1 bladder carcinomas remains uncertain due to the limited number of related studies. Most reports on HGT1 with variant histology are reporting on micropapillary carcinoma or, rarely, nested carcinoma [4, 7–20]. The fact that some pathologists do not recognize or report about one-half of cases with variant histology in their practice is an additional limitation; therefore, the risk associated with variant histology in HGT1 carcinomas might indeed be underrecognized [21, 22].

To entangle the clinical situation, the AUA (American Urological Association) risk stratification for NMIBC (non–muscle-invasive bladder cancer) includes any variant histology as high risk; and the National Comprehensive Cancer Network (NCCN) guidelines support the inclusion of NMIBC with micropapillary, plasmacytoid, and sarcomatoid morphology as high risk of progression and death and recommends aggressive therapy. Some authors support early cystectomy in T1 micropapillary carcinoma to improve cancer-specific survival (CSS) [10, 23, 24]. Nonetheless, the appropriated management of HGT1 carcinomas with variant histology remains a matter of debate. Other relevant aspects to consider include the percentage of variant histology in cases of carcinoma with mixed histology, a parameter mainly investigated in micropapillary carcinoma, and the recent data in support of squamous and lymphoepithelioma-like morphology to predict the pathological response of anti-PD-1 immunotherapy in a neoadjuvant setting relevant to the field of bladder preservation and to treat BCG-unresponsive HGT1 carcinomas [25–27]. Consequently, the ICCR dataset (International Collaboration on Cancer Reporting) recommends reporting and including an estimate of the variant histology present [6]. Thus, a risk classification associated with the different variant histology seen in HGT1 carcinoma would provide a rationale to stratify patients that can or cannot benefit from radical surgery (or other novel therapies), and therefore seems to be a necessary step of good practice in the management of these patients.

This paper aims to report the risk associated with variant histology in HGT1 bladder carcinoma compared with conventional urothelial carcinoma in a contemporary series.

## Materials and methods

A total of 104 high-grade T1 bladder carcinoma samples were retrieved from the archives of Pathology Departments of participating institutions. Clinical information was obtained from medical records, and an average of three H&E-stained slides from routine formalin-fixed and paraffin-embedded material (range, 1–6) from each case was systematically re-evaluated by three dedicated pathologists who confirmed the diagnosis and the presence of invasion to the level of subepithelial connective tissue. All cases were obtained by TURBT. Only primary cases with a negative second TURBT after the initial diagnosis were allowed. Cases with focally prominent discohesive features which occasionally imparted a plasmacytoid-like morphology were also excluded. Twelve cases were subsequently excluded upon review due to lack of relevant clinical information (seven cases lacked the applied BCG protocol or < 1-year follow-up) or the presence of features supportive of muscularis propria invasion (two cases of micropapillary, one nested, and two plasmacytoid carcinomas). The remaining 92 cases were split into two groups: (i) cases with pure urothelial morphology classified as conventional urothelial carcinoma; (ii) cases with variant histology. In the latter category, except for urothelial carcinoma with squamous or glandular divergent differentiation, ≥ 50% of variant histology was required for inclusion. The presence of concomitant urothelial carcinoma in situ and focal tumor necrosis was also included in the study. All cases received BCG (bacillus Calmette-Guerin) treatment with maintenance, following the current clinical guidelines available at time of diagnosis, or mitomycin C (7.6%). The cases spanned 14 years, with the earliest case diagnosed in 2002 and the latest in 2016, resulting in a follow-up of 13–170 months (mean ± SD, 50.14 ± 32.27). Times to event for disease recurrence and cancer mortality were calculated from the date of TURBT to the date of last follow-up or death by cancer, and survival was measured from the date of the TURBT diagnosis to the date of last follow-up or death. Histological classification and depth of invasion of the tumors followed the 2016 revisions of WHO and AJCC, respectively [28, 29]. Recurrence was defined as finding new NMIBC after complete resection and induction course with BCG, whereas progression was defined as the recurrence of a tumor with features of muscle-invasive bladder cancer after TURBT and BCG instillations.

Immunohistochemistry was performed on selected representative paraffin sections to resolve specific differential diagnostic issues required by the submitting pathologist. It included GATA3 (Cell Marque, clone L50-823, prediluted), CK20 (1:50, Dako, Glostrup, Denmark), MUC1 (Ventana, Clone H23, prediluted), p63 (1:100, Dako, Glostrup, Denmark), CK14 (Cell Marque, clone SP53, prediluted), CD3 (Cell Marque, rabbit polyclonal, prediluted), CK AE1AE3 (clone AE1/AE3, prediluted), CK-CAM 5.2 (1:2, Beckton, San Jose, CA), CK5/6 (clone 16B4; 1:50; Cell Marque, Rocklin, CA), or smoothelin (Cell Marque, clone R4A, prediluted). These were used as a single marker or as a combination thereof. Immunohistochemistry performance followed standard protocols for each antibody, using both Ventana-Benchmark or Leica Bond platforms and their respective reagents. Appropriate negative and positive controls were included in every run. Immunostaining was graded from 0 to 3 + , when appropriate.

Categorical variables were presented as frequencies and percentages and were compared using the *t*-test or chi-square test. The Kaplan–Meier method was used to estimate the distribution of survival separately for the categories with variant histology and conventional urothelial carcinoma. Differences among these two groups were tested for significance using the log-rank test (SPSS 15.0; SPSS, USA). A *P*-value ≤ 0.05 was considered statistically significant.

## Results

**Fig. 1:**
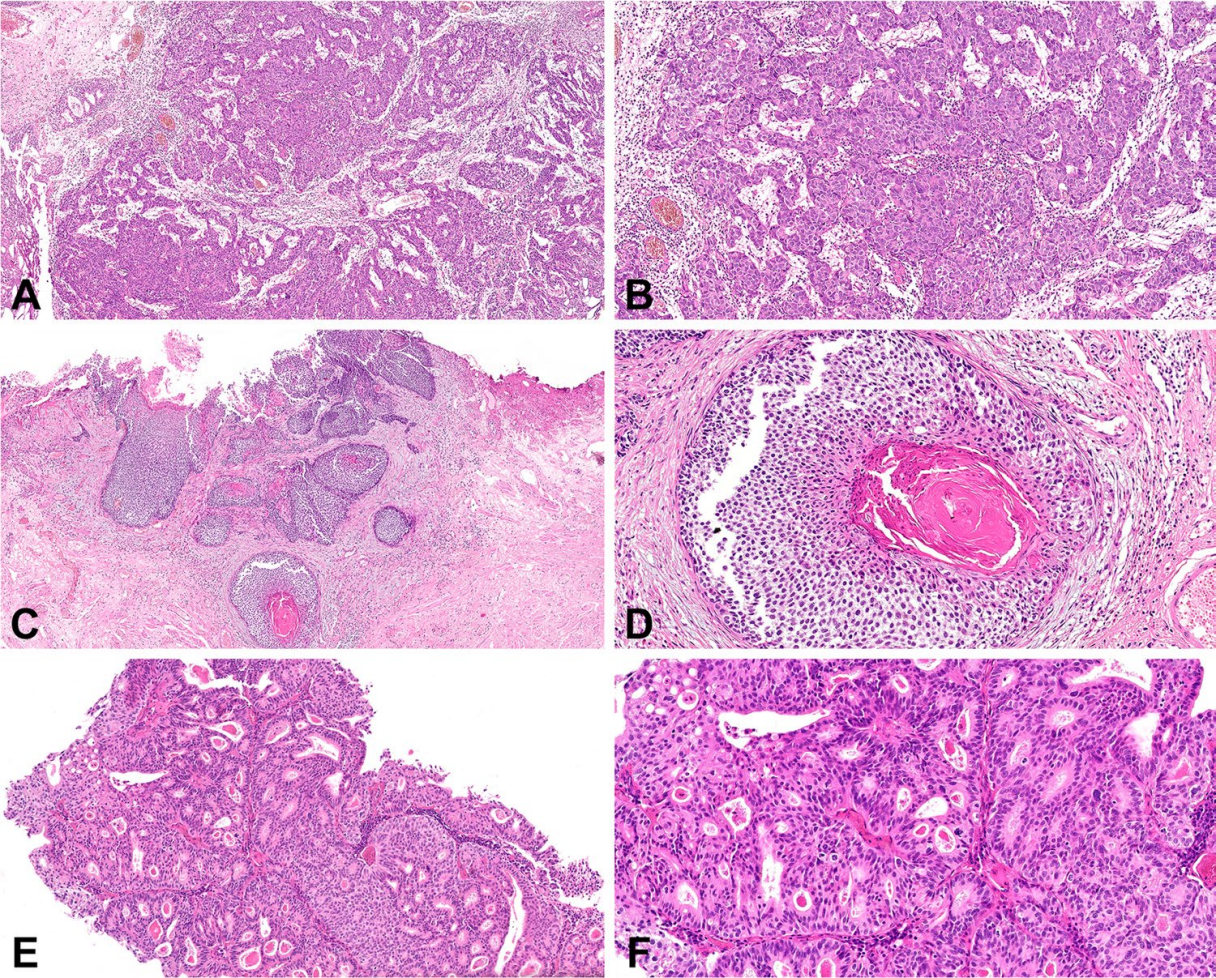
Conventional urothelial carcinoma seen at low (**a**) and high (**b**) power. Low- (**c**) and high-power (**d**) views of squamous differentiation. Glandular differentiation seen at low (**e**) and high power (**f**)

**Fig. 2:**
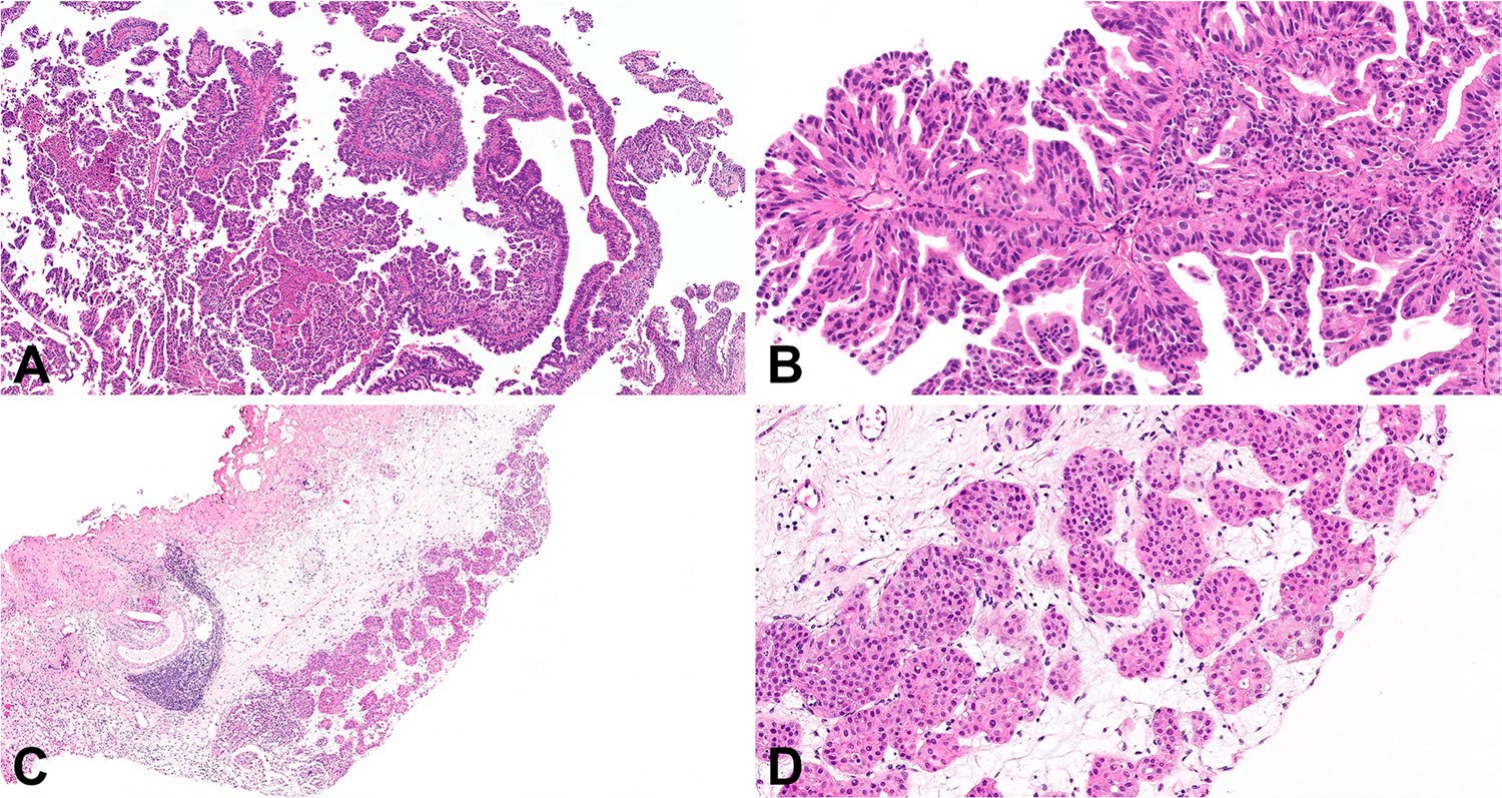
Micropapillary carcinoma seen at low (**a**) and high (**b**) power. Low- (**c**) and high-power (**d**) views of nested carcinoma

**Fig. 3:**
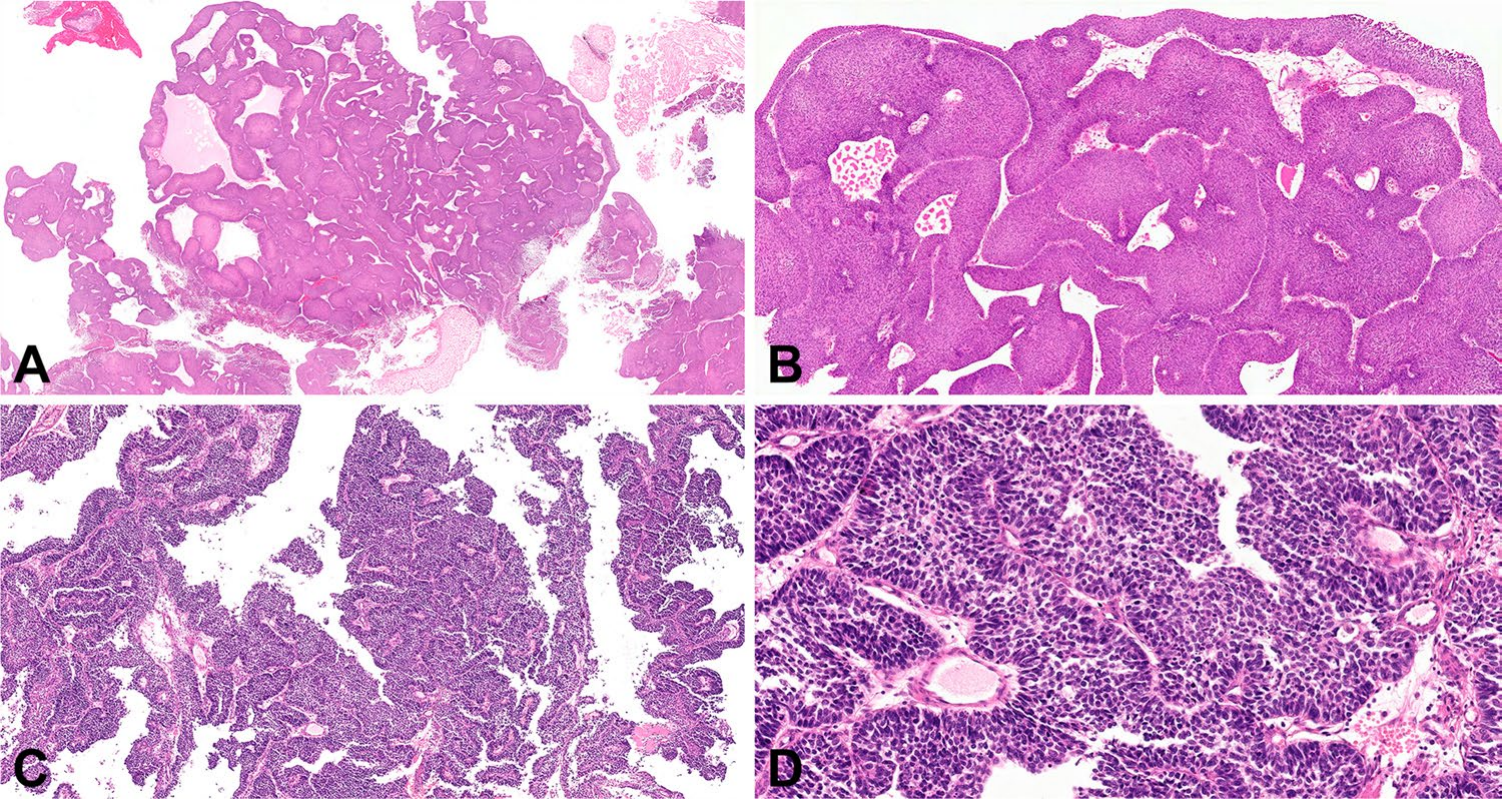
Inverted urothelial carcinoma seen at low (**a**) and high (**b**) power. Low- (**c**) and high-power (**d**) views of basaloid carcinoma

**Fig. 4:**
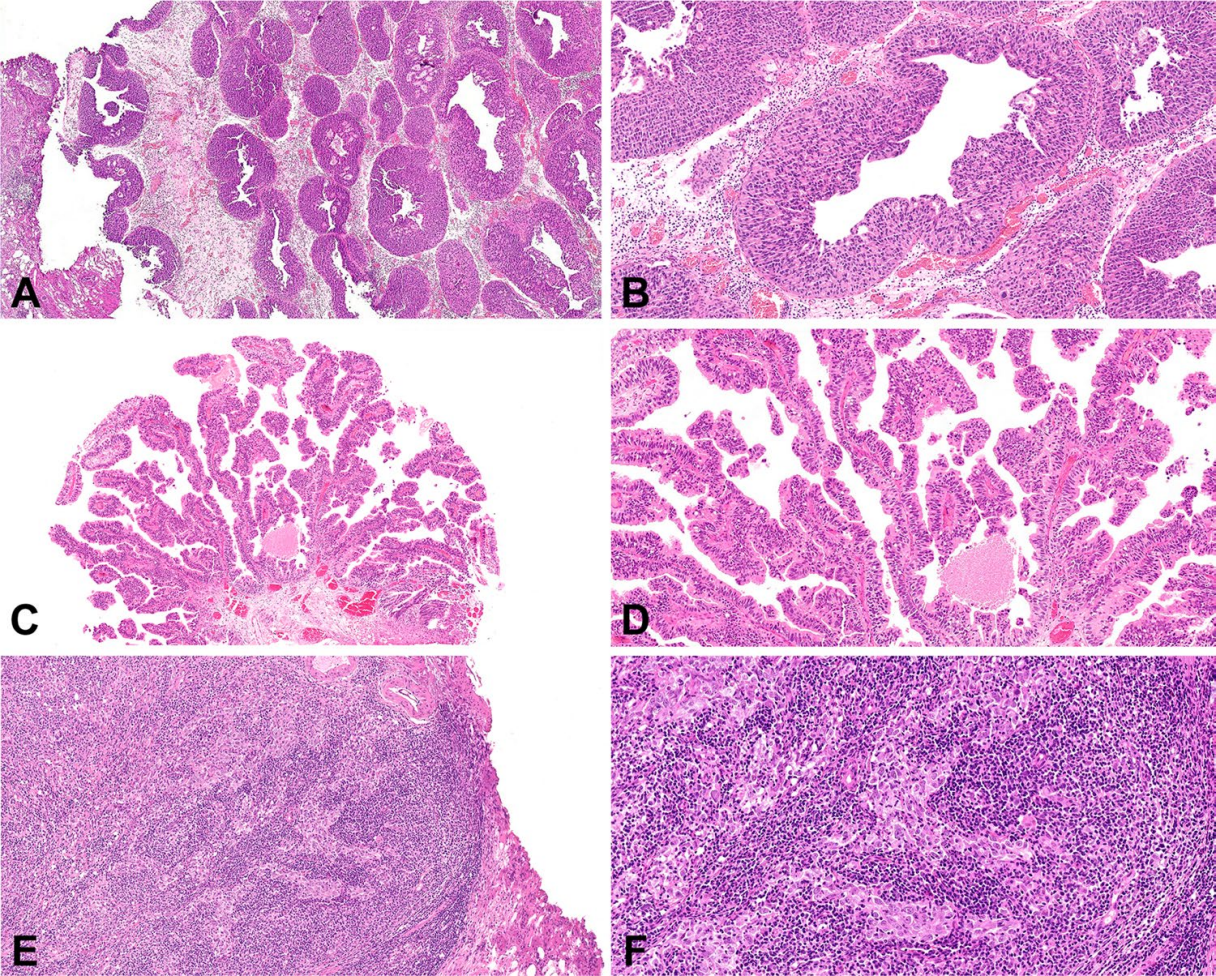
Microcystic carcinoma seen at low (**a**) and high (**b**) power. Low- (**c**) and high-power (**d**) views of villous carcinoma. Lymphoepithelioma-like seen at low (**e**) and high power (**f**)

**Fig. 5:**
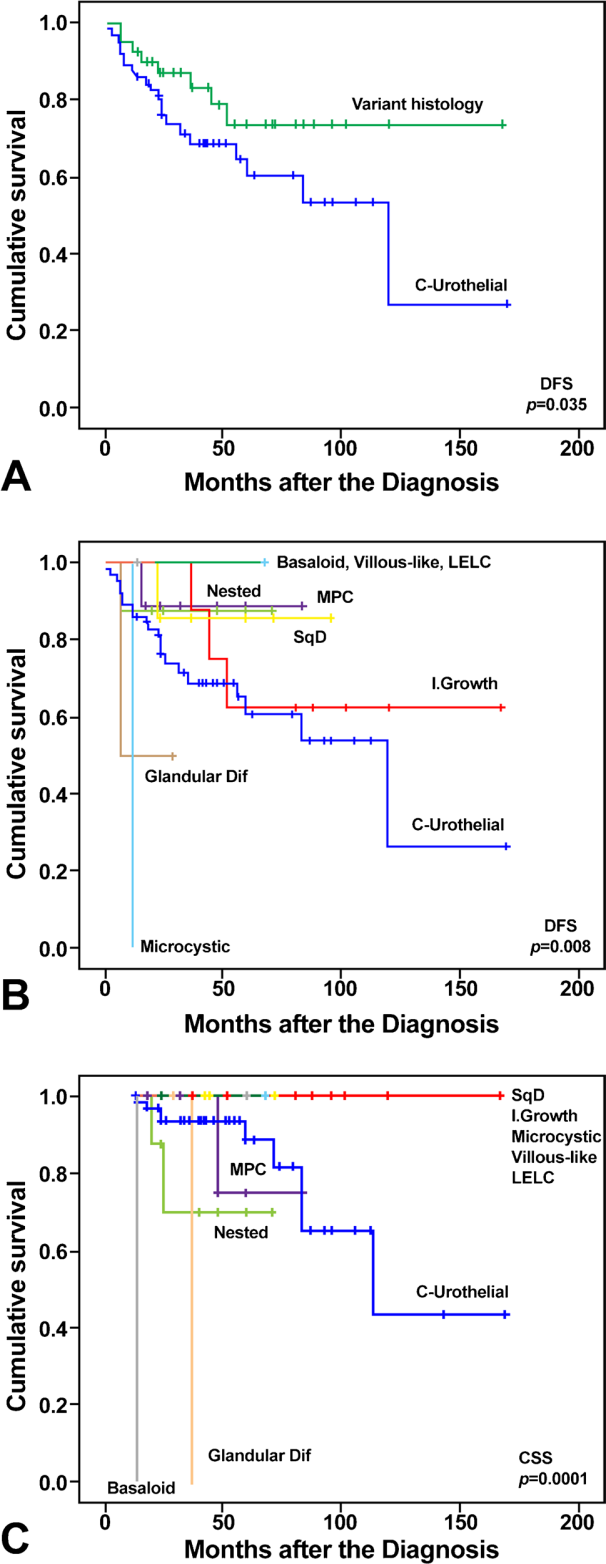
Kaplan–Meier plots identify variant histology (**a**) and variant subtypes (**b**) as significant predictors of disease-free survival (DFS) in high-grade T1 carcinoma; variant subtypes were also significant predictors of cancer-specific survival (CSS) (**c**)

**Fig. 6:**
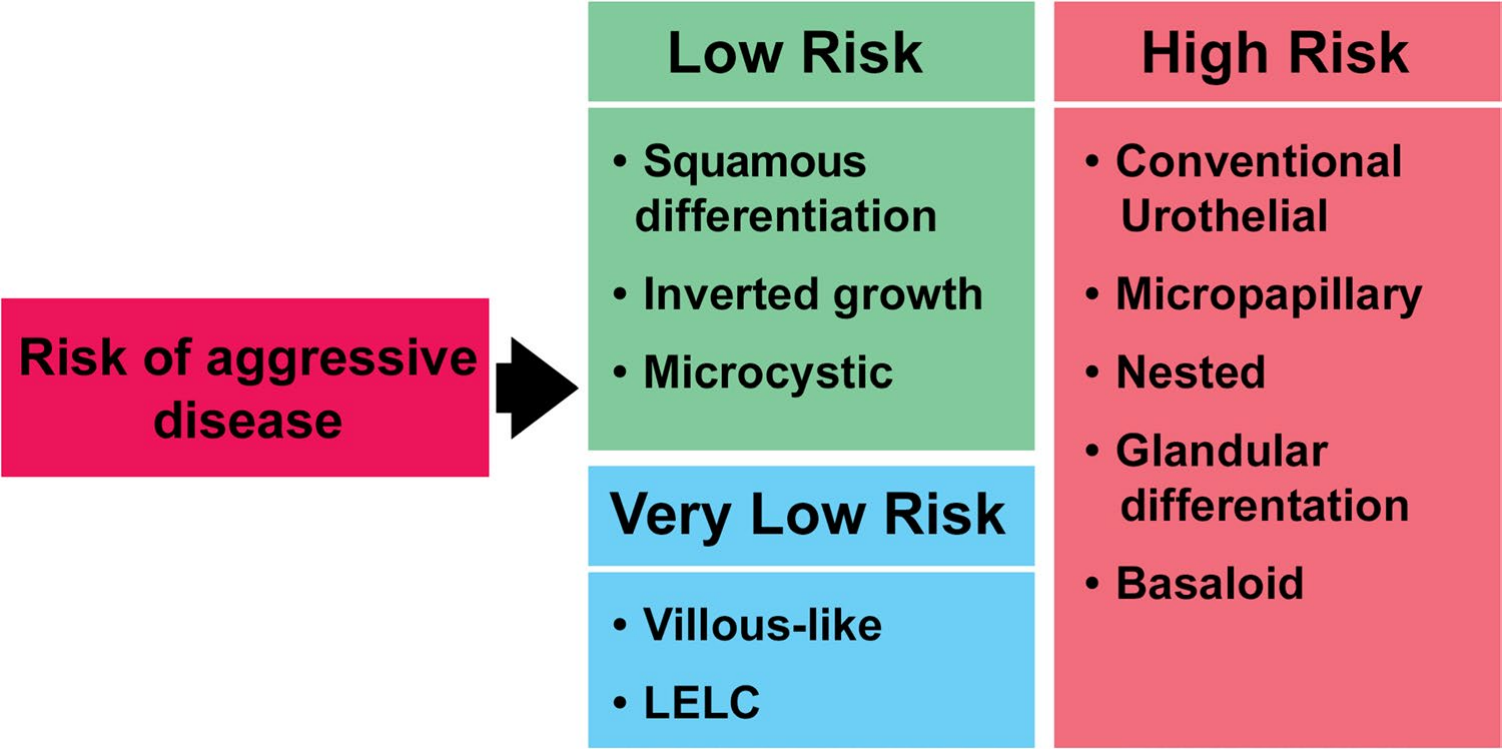
The observed risk of aggressive disease, high-grade T1 urothelial carcinoma, allows to separate variant subtypes into low- (left) and high-risk (right) categories. A very low-risk category may be considered for villous and lymphoepithelioma-like (bottom left)

The main characteristics of the 92 cases of high-grade T1 bladder carcinoma with pure urothelial morphology or with variant histology (34 cases [37%]) included nested or inverted growth carcinoma (8.7% each); micropapillary carcinoma (7.6%); divergent differentiation (squamous [5.4%] or glandular [2.2%] carcinoma; and basaloid, microcystic, villous-like, or LELC (lymphoepithelioma-like) variants (1.1% each) (Table 1; Figs. 1, 2, 3, 4). Eighty percent of patients were male with a mean age of 72.5 years (range, 38–92 years). Twenty-six (28.3%) presented with concomitant urothelial carcinoma in situ or focal tumor necrosis (15 cases [16.3%]), and 92.4% received BCG treatment. On a mean follow-up of 50 months (range, 13–170 months), 77.8%, 58.8%, and 64.3% with conventional urothelial carcinoma recurred, progressed, or died of disease, respectively. Similarly, 22.2%, 41.2%, and 35.7% with variant histology carcinoma recurred, progressed, or died of disease, respectively. Table 2 presents the univariate survival analysis in this series. There were differences between conventional urothelial carcinoma and urothelial carcinomas with variant histology (*P* = 0.035), and for specific variants (*P* = 0.008) regarding disease-free survival (DFS); and for CSS concerning variant subtype (*P* = 0.0001) (Fig. 5). Gender status, concomitant urothelial carcinoma in situ, and the presence of tumor necrosis did not reach significance regarding DFS and CSS, with borderline significance regarding the response to BCG treatment (*P* = 0.069) (Table 2). Our results allowed a risk classification of HGT1 carcinoma based on the specific variant histology present (Fig. 6).

**Table 1:** Demography and clinicopathological characteristics of the 92 cases of T1 high-grade bladder carcinoma included in the study

	***n* (%)**
**Mean age (year) ± SD (range)**	72.5 ± 9.0 (92–38)
**Mean follow-up (month) ± SD (range)**	50.1 ± 32.3 (13–170)
**Gender**	
Female	12 (13.0)
Male	80 (87.0)
**Variant histology**	
No	58 (63.0)
Yes	34 (37.0)
**Variant subtype**	
Conventional urothelial carcinoma	58 (63.0)
Nested	8 (8.7)
Glandular	2 (2.2)
Micropapillary	7 (7.6)
Squamous	5 (5.4)
Inverted	8 (8.7)
Basaloid	1 (1.1)
Microcystic	1 (1.1)
Villous-like	1 (1.1)
Lymphoepithelioma-like carcinoma	1 (1.1)
**Concomitant urothelial carcinoma in situ**	
No	66 (71.7)
Yes	26 (28.3)
**Tumor necrosis**	
No	77 (83.7)
Yes	15 (16.3)
**Bacillus Calmette-Guerin**	
No	7 (7.6)
Yes	85 (92.4)
**Tumor recurrence**	
**No**	65 (70.7)
Conventional urothelial carcinoma	37 (56.9)
Variant histology	28 (43.1)
**Yes**	27 (29.3)
Conventional urothelial carcinoma	21 (77.8)
Variant histology	6 (22.2)
**Tumor progression**	
**No**	75 (81.5)
Conventional urothelial carcinoma	48 (64.0)
Variant histology	27 (36.0)
**Yes**	17 (18.5)
Conventional urothelial carcinoma	10 (58.8)
Variant histology	7 (41.18)
**Survival status**	
**Alive**	65 (70.7)
Conventional urothelial carcinoma	39 (60.0)
Variant histology	26 (40.0)
**Dead of bladder cancer**	14 (15.2)
Conventional urothelial carcinoma	9 (64.3)
Variant histology	5 (35.7)
**Dead of other cause**	13 (14.1)

*SD*, standard deviation

**Table 2:** Univariate analysis for cancer-specific survival of parameters in the study using Kaplan–Meier plots and the Log-rank test

	**Overall *n* = 92**	**DFS**	**Log-rank**	***P*-value**	**CSS**	**Log-rank**	***P*-value**
**Gender**			0.879	0.349		0.531	0.466
Female	12	4			2		
Male	80	23			12		
**Variant histology**			4.468	0.035		0.156	0.693
No	58	21			9		
Yes	34	6			5		
**Variant subtype**			15.126	0.008		55.091	0.0001
Urothelial, not otherwise specified	58	21			9		
Nested	8	1			2		
Glandular	2	1			1		
Micropapillary	7	0			1		
Squamous	5	0			0		
Inverted	8	3			0		
Basaloid	1	0			1		
Microcystic	1	1			0		
Villous	1	0			0		
Lymphoepithelioma-like carcinoma	1	0			0		
**Bacillus Calmette-Guerin**			0.239	0.625		3.317	0.069
No	7	1			2		
Yes	85	26			12		
**Concomitant urothelial carcinoma in situ**			0.01	0.920			
No	66	20			10	0.008	0.931
Yes	26	7					
**Necrosis**			0.036	0.849		0.605	0.437
No	77	23			11		
Yes	15	4			3		

*DFS*, disease-free survival; *CSS*, cancer-specific survival

## Discussion

Bladder carcinoma is morphologically heterogeneous, a process due to intratumoral heterogeneity and ultimately reflected by the presence of the variants of bladder cancer (variant histology) [30]. The current WHO revision includes urothelial carcinoma with divergent (squamous, glandular, and trophoblastic) differentiation, nested, microcystic, micropapillary, lymphoepithelioma-like (LELC), plasmacytoid/signet ring cell/diffuse, sarcomatoid, giant cell, poorly differentiated, lipid-rich, and clear cell (glycogen-rich) as histologic variants [28]. The presence of any variant or combinations thereof is considered high risk in NMIBC; [12] the NCCN clinical guidelines also recommend immediate radical cystectomy for HGT1 with micropapillary, plasmacytoid, or sarcomatoid variants, and the AUA guidelines of NMIBC include the presence of any histologic variant as a high-risk category [31, 32]. The true incidence of variant histology in HGT1 urothelial carcinoma is uncertain, mainly based on limited reports of short case series, and ranges from 6.4 to 23% [33, 34]. Among HGT1 bladder cancer with variant histology, the micropapillary variant has received much attention, with T1 cases representing up to 40% of all-stage reported cases [9, 14, 22, 34–37]. T1 nested carcinoma represents 16% of all-stage reported cases [9, 20]. Squamous divergent differentiation is also frequently seen in HGT1 carcinomas, with a reported rate of 14% [9, 22]. Nonetheless, data concerning other variants recognized by the WHO are limited mainly to small case series in HGT1, and their actual incidence remains largely unknown [22]. The substantial interobserver discordance when the central review of diagnostic cases is performed, and the fact that most variants are not recognized in daily practice by several pathologists, may explain the limited knowledge available on the risk associated with them in practice [21, 34].

Variant histology was associated with variable aggressive clinicopathological features in the current study, including survival and recurrence rate after complete TURBT and maintenance guided BCG instillations. Interestingly, the identified variants did not all show the same risk of aggressive disease. Squamous, glandular, micropapillary, nested, microcystic, and inverted growth variants showed lower DFS than villous-like, basaloid, and LELC, which showed no recurrences. On the other hand, micropapillary, nested, glandular, and basaloid variants showed lower CSS rates than squamous, inverted, microcystic, villous-like, and LELC variants whose patients remained alive over 150 months of follow-up. Miyake et al., [12] in an extensive series of 1490 patients with NMIBC, recently reported an incidence of 6.4% with variant histology. These patients are more likely to result in cancer-related death than those with conventional urothelial carcinoma or with divergent differentiation, similar to our findings concerning risk stratification; in fact, our cases with divergent squamous differentiation were included as part of the low-risk category, and cases such as micropapillary and nested carcinomas remained as aggressive, life-threatening high-risk diseases. Likewise, Vourganti et al. [38], using data from the Surveillance, Epidemiology, and End Results (SEER) database, found no survival difference for micropapillary compared to conventional urothelial carcinoma after adjusting for stage and grade. Interestingly, these authors found similar behavior for cases reported as low-grade micropapillary NMIBC compared with high-grade micropapillary and high-grade urothelial carcinoma. This is an interesting point of practice, and a potential pitfall since rare cases of T1 micropapillary carcinomas may have bland cytology, and one may wonder if they should be categorized as low grade [38].

Currently, most publications on T1 urothelial carcinoma with variant histology have been dedicated to micropapillary or, rarely, to nested variants. However, our study additionally included a representation of other variants seen in HGT1 carcinomas with different sensitivities to recurrence and survival. This approach allowed us to identify a low- vs. high-risk classification associated with different variants. This includes micropapillary and nested, both reportedly associated with aggressive behavior [2, 35]. Consequently, our results support the inclusion of HGT1 carcinoma with micropapillary or nested features within the high-risk (aggressive) category. However, caution should be given to managing patients with micropapillary carcinomas since some studies suggest that these may be well controlled using standard TURBT and BCG therapy [14]. Therefore, it remains controversial if NMIBC with micropapillary features should undergo aggressive therapy ab initio due to some studies in which limited representation of superficial micropapillary features (less than 10% micropapillary) was unrelated to aggressive behavior compared with conventional urothelial carcinoma; these cases should not be classified as micropapillary carcinoma [1, 15, 19, 25, 28, 37].

Our study also supports a subgroup of variants with low-risk features, which also agrees with the limited data reported in the current literature. The prognostic significance of variant histology in HGT1 tumors remains controversial; however, we hypothesized that our high-risk category follows similar significance as to the reported for variant histology in advanced bladder carcinomas in which variant histology seems to be related to poor behavior after current therapies. Our study, therefore, confirms the potential value of variant histology in risk assessment of HGT1 bladder cancer through different sensitivities regarding recurrence, progression, or survival associated with different histologic variants (see graphic representation, Fig. 6), although, our risk categorization is mostly based on cancer specific survival following guidelines supported therapy. A challenging point is that there were no cases of plasmacytoid carcinoma in our series, similar to Miyake’s recent study of 1490 cases of HGT1 carcinoma; this is probably due to the rarity of the histologic variant in HGT1 carcinomas, with no reported series to date in the English literature [12, 39]. It seems reasonable to include plasmacytoid morphology as part of our high-risk category.

The retrospective nature of the current study and the fact that different pathologists selected the cases in different institutions with only one case each of the basaloid, microcystic, villous-like and lymphoepithelioma-like variants should be considered a limitation. However, our pathology-oriented series represents histologic variants other than those reported in clinically oriented series mainly limited to micropapillary and, to a lesser extent, to nested carcinomas [35, 40]. This is interesting since, clinical series exemplify the confusion associated with histologic variants by some clinical groups, but also exemplify the differences in reporting criteria from pathologists in different institutions; for instance, some of the reported series included only the presence of squamous or glandular divergent differentiation, while others included only micropapillary or nested variants with the concept of variants [15, 35, 41]. To avoid entity-derived limitations, we have conducted an accurate review of all pathologic materials, following classic diagnostic criteria for urothelial carcinomas updated to include the terminology and subtypes recognized by the current WHO classification of urogenital tumors, as well as recently published data [1, 3, 28]. Additionally, our cases were diagnosed based on specialized genitourinary pathological assessment using contemporary criteria, which can also explain the inclusion of some rarer examples of variant histology not recognized by the current WHO classification (inverted growth, villous-like and basaloid carcinomas) [42]. In a context in which pathologic prognostic assessment is essential in HGT1 carcinoma management, reporting the presence and extension (i.e., percentage) of histologic variants seems to be essential as an element of good clinical practice.

In conclusion, we were able to identify a series of high-grade T1 bladder carcinomas in which the presence of specific variants offered further predictive information in this highly heterogeneous tumor, and that the disease control is likely achieved with a combination of complete TURBT and BCG therapy applied following the current guidelines. Consequently, patients with micropapillary, nested, or basaloid morphology or glandular divergent differentiation carcinoma should be considered in the context of the high-risk disease since they are potentially life-threatening progressive tumors with variable-to-limited responses to therapy. The presence of divergent squamous differentiation, inverted growth, microcystic, and villous-like or LELC morphology should be considered within the low-risk disease with variable tumor recurrence and very low progression to life-threatening disease. If confirmed in a more extensive series or well-controlled clinical trials, our variant histology proposed risk stratification could be practice-changing in managing HGT1 urothelial carcinomas.
